# Supplementary material for: The Potential Release of Chemicals from Crumb Rubber Infill Material—A Literature Review
Source: J Xenobiot. 2025 Oct 2;15(5):159. doi: 10.3390/jox15050159 (PMC12565206; doi:10.3390/jox15050159)
Supplement: Supplementary file 1 [file jox-15-00159-s001.zip › supplementary/Supplementary S.1 (Search strings and Abbreviations).pdf]

The Bioaccessibility of Chemicals in Crumb Rubber Infill Material. A Literature Review.

Supplementary S.1

Search Strings

PubMed

#1 "artificial turf*"[tiab] OR "synthetic turf*"[tiab] OR "artificial grass"[tiab] OR "synthetic grass"[tiab]
#2 "crumb rubber"[tiab] OR "tire crumb"[tiab] OR "tyre crumb"[tiab:~0] OR "shredded tire*"[tiab] OR "shredded tyre*"[tiab] OR "recycled tire*"[tiab] OR "recycled tyre*"[tiab] OR "scrap tire*"[tiab] OR "scrap tyre*"[tiab] OR "tire wear"[tiab] OR "tyre wear"[tiab] OR "poured rubber"[tiab] OR "rubber granulate*"[tiab] OR "rubber granule*"[tiab] OR "tire rubber"[tiab] OR "tyre rubber"[tiab] OR "tire particle*"[tiab] OR "tyre particle*"[tiab] OR "tire debris"[tiab] OR "tyre debris"[tiab:~0]
#3 ("Parks, Recreational"[Mesh] OR playground*[tiab] OR "play area*"[tiab]) AND ("Rubber"[Mesh] OR rubber[tiab] OR infill[tiab] OR tire[tiab] OR tires[tiab] OR tyre[tiab] OR tyres[tiab])
#4 #1 OR #2 OR #3
#5 "Health"[Mesh] OR "Public Health"[Mesh] OR "Health Surveys"[Mesh] OR health*[tiab]
#6 "toxicity"[subheading] OR "Toxicity Tests"[MeSH] OR toxic*[tiab] OR neurotoxic*[tiab] OR cytotoxic*[tiab] OR reprotoxic*[tiab] OR genotoxic*[tiab] OR immunotoxic*[tiab]
#7 "Ecotoxicology"[Mesh] OR ecotoxic*[tiab] OR toxin*[tiab]
#8 "adverse effects"[subheading]
#9 "Risk Assessment"[Mesh] OR risk*[tiab]
#10 "Hazardous Substances"[MeSH] OR hazard*[tiab] OR harm*[tiab] OR concern[tiab] OR concerns[tiab]
#11 "Carcinogens, Environmental"[Mesh] OR carcinogen*[tiab]
#12 "Mutagens"[Mesh] OR mutagen*[tiab]
#13 "Environmental Exposure"[MeSH] OR "Environmental Health"[Mesh] OR "Environmental Monitoring"[Mesh] OR expos*[tiab] OR monitor*[tiab] OR surveill*[tiab]
#14 "Incidence"[Mesh] OR incidence[tiab]
#15 "Prevalence"[Mesh] OR prevalence[tiab]
#16 "epidemiology"[Subheading] OR epidemiol*[tiab]
#17 "Mortality"[Mesh] OR mortality[tiab]
#18 bio-accessibility[tiab] OR bioaccessibility[tiab]
#19 "Fluids and Secretions"[Mesh] OR bio-fluid*[tiab] OR biofluid*[tiab] OR blood[tiab] OR saliva[tiab] OR sweat[tiab] OR tears[tiab] OR urine[tiab]
#20 #5 OR #6 OR #7 OR #8 OR #9 OR #10 OR #11 OR #12 OR #13 OR #14 OR #15 OR #16 OR #17 OR #18 OR #19
#21 #4 AND #20

Embase

#1 'artificial turf'/exp OR 'artificial turf*':ti,ab,kw OR 'synthetic turf*':ti,ab,kw OR 'artificial grass':ti,ab,kw OR 'synthetic grass':ti,ab,kw

#2 'crumb rubber'/exp OR 'crumb rubber':ti,ab,kw OR 'tire crumb':ti,ab,kw OR 'tyre crumb':ti,ab,kw OR 'shredded tire*':ti,ab,kw OR 'shredded tyre*':ti,ab,kw OR 'recycled tire*':ti,ab,kw OR 'recycled tyre*':ti,ab,kw OR 'scrap tire*':ti,ab,kw OR 'scrap tyre*':ti,ab,kw OR 'tire wear particle'/exp OR 'tire wear':ti,ab,kw OR 'tyre wear':ti,ab,kw OR 'poured rubber':ti,ab,kw OR 'rubber granulate*':ti,ab,kw OR 'rubber granule*':ti,ab,kw OR 'tire rubber':ti,ab,kw OR 'tyre rubber':ti,ab,kw OR 'tire particle*':ti,ab,kw OR 'tyre particle*':ti,ab,kw OR 'tire debris':ti,ab,kw OR 'tyre debris':ti,ab,kw

#3 ('recreational park'/exp OR playground*:ti,ab,kw OR 'play area*':ti,ab,kw) AND ('rubber'/exp OR 'motor vehicle tire'/exp OR rubber:ti,ab,kw OR infill:ti,ab,kw OR tire:ti,ab,kw OR tires:ti,ab,kw OR tyre:ti,ab,kw OR tyres:ti,ab,kw)

#4 #1 OR #2 OR #3

#5 'health'/exp OR 'public health'/exp OR 'epidemiological surveillance'/exp OR health*:ti,ab,kw

#6 'toxicity'/exp OR 'toxicity testing'/exp OR toxic*:ti,ab,kw OR neurotoxic*:ti,ab,kw OR cytotoxic*:ti,ab,kw OR reprotoxic*:ti,ab,kw OR genotoxic*:ti,ab,kw OR immunotoxic*:ti,ab,kw

#7 'ecotoxicology'/exp OR ecotoxic*:ti,ab,kw OR toxin*:ti,ab,kw

#8 'risk assessment'/exp OR risk*:ti,ab,kw

#9 'hazard'/exp OR hazard*:ti,ab,kw OR harm*:ti,ab,kw OR concern:ti,ab,kw OR concerns:ti,ab,kw

#10 'carcinogen'/de OR carcinogen*:ti,ab,kw

#11 'mutagenic agent'/de OR mutagen*:ti,ab,kw

#12 'exposure'/exp OR 'environmental health'/exp OR 'environmental monitoring'/exp OR expos*:ti,ab,kw OR monitor*:ti,ab,kw OR surveill*:ti,ab,kw

#13 'incidence'/exp OR incidence:ti,ab,kw

#14 'prevalence'/exp OR prevalence:ti,ab,kw

#15 epidemiology:lnk OR epidemiol*:ti,ab,kw

#16 'mortality'/exp OR mortality:ti,ab,kw

#17 bio-accessibility:ti,ab,kw OR bioaccessibility:ti,ab,kw

#18 'biofluid'/exp OR 'body fluids and secretions'/exp OR bio-fluid*:ti,ab,kw OR biofluid*:ti,ab,kw OR blood:ti,ab,kw OR saliva:ti,ab,kw OR sweat:ti,ab,kw OR tears:ti,ab,kw OR urine:ti,ab,kw

#19 #5 OR #6 OR #7 OR #8 OR #9 OR #10 OR #11 OR #12 OR #13 OR #14 OR #15 OR #16 OR #17 OR #18

#20 #4 AND #19

CAB Direct

#1 title:("artificial turf*" OR "synthetic turf*" OR "artificial grass" OR "synthetic grass" OR "crumb rubber" OR "tire crumb" OR "tyre crumb" OR "shredded tire*" OR "shredded tyre*" OR "recycled tire*" OR "recycled tyre*" OR "scrap tire*" OR "scrap tyre*" OR "tire wear" OR "tyre wear" OR "poured rubber" OR "rubber granulate*" OR "rubber granule*" OR "tire rubber" OR "tyre rubber" OR "tire particle*" OR "tyre particle*" OR "tire debris" OR "tyre debris") OR ab:("artificial turf*" OR "synthetic turf*" OR "artificial grass" OR "synthetic grass" OR "crumb rubber" OR "tire crumb" OR "tyre crumb" OR "shredded tire*" OR "shredded tyre*" OR "recycled tire*" OR "recycled tyre*" OR "scrap tire*" OR "scrap tyre*" OR "tire wear" OR "tyre wear" OR "poured rubber" OR "rubber granulate*" OR "rubber granule*" OR "tire rubber" OR "tyre rubber" OR "tire particle*" OR "tyre particle*" OR "tire debris" OR "tyre debris")

#2 subject:("playgrounds") OR title:(playground* OR "play area*") OR ab: :(playground* OR "play area*")

#3 subject:(rubber OR tyres) OR title:(rubber OR infill OR tire OR tires OR tyre OR tyres) OR ab:(rubber OR infill OR tire OR tires OR tyre OR tyres)

#4 #2 AND #3

#5 #1 OR #4

#6 subject:("health" OR "public health") OR title:(health*) OR ab:(health*)

#7 subject:("toxicity") OR title:(toxic* OR neurotoxic* OR cytotoxic* OR reprotoxic* OR genotoxic* OR immunotoxic* OR ecotoxic* OR toxin*) OR ab:(toxic* OR neurotoxic* OR cytotoxic* OR reprotoxic* OR genotoxic* OR immunotoxic* OR ecotoxic* OR toxin*)

#8 subject:("adverse effects")

#9 subject:("risk assessment") OR title:(risk*) OR ab:(risk*)

#10 subject:("hazards") OR title:(hazard* OR harm* OR concern OR concerns) OR ab:(hazard* OR harm* OR concern OR concerns)

#11 subject:("carcinogens") OR title:(carcinogen*) OR ab:(carcinogen*)

#12 subject:("mutagens") OR title:(mutagen*) OR ab:(mutagen*)

#13 subject:("exposure" OR "environmental health") OR title:(expos* OR monitor* OR surveill*) OR ab:(expos* OR monitor* OR surveill*)

#14 subject:("incidence") OR title:(incidence) OR ab:(incidence)

#15 subject:("disease prevalence") OR title:(prevalence) OR ab:(prevalence)

#16 subject:("epidemiology") OR title:(epidemiol*) OR ab:(epidemiol*)

#17 subject:("mortality") OR title:(mortality) OR ab:(mortality)

#18 title:("bio-accessibility" OR bioaccessibility) OR ab:("bio-accessibility" OR bioaccessibility)

#19 subject:("body fluids" OR "secretions") OR title:("bio-fluid*" OR biofluid* OR blood OR saliva OR sweat OR tears OR urine) OR ab:("bio-fluid*" OR biofluid* OR blood OR saliva OR sweat OR tears OR urine)

#20 #6 OR #7 OR #8 OR #9 OR #10 OR #11 OR #12 OR #13 OR #14 OR #15 OR #16 OR #17 OR #18 OR #19

#21 #5 AND #20

Scopus

#1 TITLE-ABS-KEY("artificial turf*" OR "synthetic turf*" OR "artificial grass" OR "synthetic grass")
#2 TITLE-ABS-KEY("crumb rubber" OR "tire crumb" OR "tyre crumb" OR "shredded tire*" OR "shredded tyre*" OR "recycled tire*" OR "recycled tyre*" OR "scrap tire*" OR "scrap tyre*" OR "tire wear" OR "tyre wear" OR "poured rubber" OR "rubber granulate*" OR "rubber granule*" OR "tire rubber" OR "tyre rubber" OR "tire particle*" OR "tyre particle*" OR "tire debris" OR "tyre debris")
#3 TITLE-ABS-KEY(playground* OR "play area*") AND TITLE-ABS-KEY(rubber OR infill OR tire OR tires OR tyre OR tyres)
#4 #1 OR #2 OR #3
#5 TITLE-ABS-KEY(health*)
#6 TITLE-ABS-KEY(toxic* OR neurotoxic* OR cytotoxic* OR reprotoxic* OR genotoxic* OR immunotoxic* OR ecotoxic* OR toxin*)
#7 TITLE-ABS-KEY(risk*)
#8 TITLE-ABS-KEY(hazard* OR harm* OR concern OR concerns)
#9 TITLE-ABS-KEY(carcinogen* OR mutagen*)
#10 TITLE-ABS-KEY(expos* OR monitor* OR surveill*)
#11 TITLE-ABS-KEY(incidence OR prevalence OR epidemiol* OR mortality)
#12 TITLE-ABS-KEY(bio-accessibility OR bioaccessibility)
#13 TITLE-ABS-KEY(bio-fluid* OR biofluid* OR blood OR saliva OR sweat OR tears OR urine)
#14 #5 OR #6 OR #7 OR #8 OR #9 OR #10 OR #11 OR #12 OR #13
#15 #4 AND #14
#16 INDEX(Medline OR Embase)
#17 PMID(1* OR 2* OR 3* OR 4* OR 5* OR 6* OR 7* OR 8* OR 9* OR 0*)
#18 #16 OR #17
#19 #15 AND NOT #18

Web of Science

#1 TS=("artificial turf*" OR "synthetic turf*" OR "artificial grass" OR "synthetic grass")
#2 TS=("crumb rubber" OR "tire crumb" OR "tyre crumb" OR "shredded tire*" OR "shredded tyre*" OR "recycled tire*" OR "recycled tyre*" OR "scrap tire*" OR "scrap tyre*" OR "tire wear" OR "tyre wear" OR "poured rubber" OR "rubber granulate*" OR "rubber granule*" OR "tire rubber" OR "tyre rubber" OR "tire particle*" OR "tyre particle*" OR "tire debris" OR "tyre debris")
#3 TS=(playground* OR "play area*") AND TS=(rubber OR infill OR tire OR tires OR tyre OR tyres)
#4 #1 OR #2 OR #3
#5 TS=health*
#6 TS=(toxic* OR neurotoxic* OR cytotoxic* OR reprotoxic* OR genotoxic* OR immunotoxic* OR ecotoxic* OR toxin*)
#7 TS=risk*
#8 TS=(hazard* OR harm* OR concern OR concerns)
#9 TS=(carcinogen* OR mutagen*)
#10 TS=(expos* OR monitor* OR surveill*)
#11 TS=(incidence OR prevalence OR epidemiol* OR mortality)
#12 TS=(bio-accessibility OR bioaccessibility)
#13 TS=(bio-fluid* OR biofluid* OR blood OR saliva OR sweat OR tears OR urine)
#14 #5 OR #6 OR #7 OR #8 OR #9 OR #10 OR #11 OR #12 OR #13
#15 #4 AND #14.

Abbreviations

6PPD = N-(1,3-Dimethylbutyl)-N′-phenyl-p-phenylenediamine; 6PPD-quinone = N-(1,3-Dimethylbutyl)-N′-phenyl-p-phenylenediamine-quinone; Ag = Silver; Al = Aluminium; As = Arsenic; B[α]P = Benzo[a]pyrene; Ba = Barium; Be = Beryllium; BPA = Bisphenol A; BTZ = Benzothiazole; Cd = Cadmium; Co = Cobalt; Cr = Chromium; Cu = Copper; DAD = Diode Array Detection; DI-SPME = Direct Solid Phase Microextraction; ECHA = European Chemicals Agency; ELTs = End-of-Life Tyres; EPA = Environmental Protection Agency; EPDM = Ethylene Propylene Diene Monomer; Fe = Iron; GC-MS = Gas Chromatography coupled with Mass Spectrometry; Hg = Mercury; HMMM = hexamethoxymethylmelamine; HPLC = High-Performace Liquid Chromatography; ICP-MS = Inductively Coupled Plasma Mass Spectrometery; LOD = Limit of Detection; LOQ = Limit of Quantification; MBTZ = 2-Mercaptobenzothiazole; Mg = Magnesium; MIBK = Methyl Isobutyl Ketone; Mn = Manganese; Ni = Nickel; NR = Natural Rubber; OEHHA = California Office of Environmental Health Hazard Assessment; PAHs = Polycyclic Aromatic Hydrocarbons; PCBs = Polychlorinated Biphenyls; PCTs = Post-Consumer Tyres; Pb = Lead; PHN = Phenanthrene; REACH = Registration, Evaluation, Authorisation and Restriction of Chemicals; RIVM = National Institute for Public Health and the Environment; Sb = Antimony; SBR = Styrene Butadiene Rubber; Se = Selenium; SPE = Solid-Phase Extraction; Sr = Strontium; SVOCs = Semi-Volatile Aromatic Hydrocarbons; Tl = Thallium; TPE = Thermoplastic Elastomer; UAE = Ultrasound-Assisted Extraction; V = Vanadium; VOCs = Volatile Organic Compounds; WHO = World Health Organisation; Zn = Zinc.
