# Supplementary material for: The Potential Release of Chemicals from Crumb Rubber Infill Material—A Literature Review
Source: J Xenobiot. 2025 Oct 2;15(5):159. doi: 10.3390/jox15050159 (PMC12565206; doi:10.3390/jox15050159)
Supplement: Supplementary file 1 [file jox-15-00159-s001.zip › supplementary/Supplementary S.2 (Tables) - updated.pdf]

Artificial Turf and Human Health: Potential Bioaccessibility of Chemicals in Crumb Rubber and (Non-)Carcinogenic Risks Associated with Their Exposure. A Literature Review.

Table S1 - Studies reporting bioaccessibility data in crumb rubber infill material

Reference	Country	Matrix	Age of the pitch and particle size	Markers	Analytical Methods	Biofluid	Quantification Methods	Main Findings
				Scientific literature				
**Armada et al. (2023) [31]**	Spain	Crumb rubber infill from different pitches across Europe	3 months to 6 years + new commercial samples; 1.20 ± 0.26 mm to 2.79 ± 1.00 mm	PAHs	*Biofluids*: UBM followed by SPE; *PAHs in crumb rubber*: UAE	Saliva<br>Gastric Juice<br>Duodenal Juice<br>Bile	GC–MS/MS	15/16 EPA PAHs were found in gastrointestinal fluids;<br>Volatile PAH shows the highest bioaccessibility rate;<br>Compounds reported for the first time in digestive biofluids: 6PPD, 6PPD-quinone, HMMM, BTZ, MBTZ.
**Kawakami et al. (2022) [32]**	Japan	Crumb rubber	new samples	Vulcanisation accelerators, Antioxidants, Decomposition products of curing agents, Vulcanisation retarders, Peptising agents, Plasticisers, Light stabilisers, Others	*Biofluids: LC-MS/MS Chemicals in crumb rubber: Ultrasonic exctraction*	Saliva<br>Gastric Juice<br>Intestinal Juice<br>Acidic sweat	*LC-MS/MS*	27 compounds detected;<br>MBT, ETU and AP showed an elution rate < 10%, in contrast with BTZ, BZL, TEP, and PI showing up to 90%.<br>Plasticisers revealed an elution rate <LOQ. The higher elution rates were measured in simulated gastric juices, expecially for antioxidants.

**Kim et al. (2012b) [33]**	Korea	EPDM	n.a.;<br>> 250 µm or < 250 µm	Pb	*Biofluids*: Artificial digestive extraction;<br>*Pb in crumb rubber*: Total content test (microwave digestion) and Acid extraction	Gastric Juice<br>Duodenal Juice	ICP-MS	The acid extraction resulted in higher bioavailability fractions than digestive extraction;<br>Pb was ten times higher in EPDM than in the digestive extract;<br>Bioavailability was higher in smaller particles (< 250 µm).
**Kim et al. (2012a) [34]**	Korea	Rubber chips	n.a;<br>n.a.	Metals	*Total content* Acid extraction<br>*Biofluids*: Digestive extraction	Gastric Solution	ICP-MS	Pb and Cr extracted by gastric solutions resulted in concentrations 10 times higher than in the acid extract;<br>Pb bioavailability: up to 41%<br>Cr bioavailability: up to 9%.
**Kubota et al. (2022) [35]**	Japan	EPDM, SBR, NBR, TPE, NR	(New samples);<br>n.a.	Metals	*Biofluids:* Biofluid extraction;<br>*Metals in crumb rubber:* microwave-assisted acid digestion	Saliva<br>Gastric Fluid<br>Intestinal Fluid<br>Sweat	ICP-MS	16 metals were detected in at least one biofluid;<br>The highest concentrations were detected in artificial gastric fluid.
**Luo et al. (2025) [36]**	China	Crumb rubber	Different ages: 1 year, 5 years, and 10 years	Metals	*Biofluids:* SBRC	Gastrointestinal fluid	ICP-MS	Zn, As, and Cd decreased from the oldest to the newest, and was higher in samples from the northern regions.<br>Gastric phase: As bioaccessibility > 0.20 in samples with a construction time of ten years and form the northern

								regions; Zn, As, and Cd bioaccessibility < 0.20 from samples with a lower construction time.<br>Intestinal phase: As bioaccessibility ranging between 0.20 and 0.40.
**Nishi et al. (2022) [37]**	Japan	Discarded tires, industrial rubber, EPDM	(New samples); 0.5 – 3.2 mm	PAHs and related compounds	*Biofluids:*<br>Elution test<br>*PAHs in rubber samples*:<br>ultrasonic extraction	Saliva<br>Gastric Juice<br>Intestinal Juice<br>Sweat	GC-MS	No PAHs above the limit of quantification (LOQ) were detected.
**Pavilonis et al. (2014) [38]**	USA	Crumb rubber infill and turf fibers	(New samples); n.a.	SVOCs, Metals	*Biofluids and total extractable fraction*:<br>SVOCs: DI-SPME<br>Metals: Nitric acid microwave digestion	Saliva<br>Gastric Juice<br>Intestinal Juice<br>Sweat<br>Lung Solution	SVOCs: GC/MS<br>Metals: ICP/MS	PAHs routinely below LODs in all biofluids. The SVOCs bioaccessibility was the highest in sweat and the lowest in digestive fluids;<br>The majority of the metals investigated were below LOD. Pb, Ti, and V were the most common, with Pb detected in almost all samples and in both digestive fluids and sweat.
**Pronk et al. (2020) [14]**	Netherland	Infill	n.a.; n.a.	VOCs, PAHs, Phthalates, Metals, Others	*Biofluids*:<br>Migration test	Evaporated Air<br>Saliva<br>Gastrointestinal Juice<br>Sweat	GC-MS	VOCs below the LOD or at very low concentrations;<br>PAHs and metals were found into gastric/intestinal fluids and sweat;<br>Phthalates migrated only into gastrointestinal fluids.

**Schneider et al. (2020) [39]**	Germany	Turf samples	n.a; n.a.	PAHs, Phthalates, Benzothiazoles and amines; BPA; others	*Biofluids*: Migration test Phthalates: SPE	Saliva Gastric Juice Sweat	PAHs and others: GC-MS Phthalates and BPA: SPE > GC-MSD Benzothiazoles and amines: HPLC-DAD Metals: ICP-MS	High variability in bioaccessibility; None of the 8 REACH PAHs was detected in artificial sweat in concentration above the LOQs. MIBK and 4-*tert*-octylphenol were found in all the artificial matrices investigated Migration to artificial sweat was also detected for BPA and DINP.
**Soñora et al. (2024) [40]**	Spain	Crumb rubber	New; n.a.	Others	*Crumb rubber:* SPE *Biofluids:* UBM	Saliva; Gastric juice; Duodenal juice; Bile	LC-MS/MS	DMBA, 6PPDq and BTZ were measurable in all the biofluids, this last up to 330 µgL-1. % Bioaccessibility: BTZ 20-50%; DMBA 20%, DPG 16%, MBTZ 13%, DMBA 10%, CBS 5.6%, DTG 5%, IPPD 4.9%, 6PPDq 1.8%, DPPD 0.6%, and 6PPD 0.2%.
**Tian et al. (2024) [41]**	China	Dust samples from outdoor sports courts	n.a.	Metals	*Crumb rubber:* spICP-TOF-MS*; Biofluids:*	Gastric juices Sweat	ICP-MS; HPLC-ICP-MS	Bioavailability data were presented as aggregated and not referred only to synthetic turf. Gastric juice is more effective in enhancing the leaching process, with concentrations ~5-25 times higher than in sweat. The prolonged exposure to dust is related to a higher bioavailability of these compounds. The concentrations in simulated biofluids were 1-2 times higher than in simulated rainwater.

								Among the metal(loid)s under investigation, in simulated sweat exposed to dust from was re-trieved the highly toxic As (III), probably due to blue/green pigments containing Cu-As.
**Winz et al. (2023) [42]**	USA	Tire crumb rubber tiles	New	Metals	*Crumb rubber and Biofluids:* microwave digestion	Filter papers to simulate children hand touching	ICP-MS	The measured concentration was positively correlated with the concentration in the crumb rubber; Pb showed a bulk content ~3 times higher than Ba and Cr, but a surface release ~4 times lower. Colour additives were not found to be determining.
**Zhang et al. (2008) [43]**	USA	Artificial turf infill	2 months – 7 years; n.a.	PAHs and Metals	*Biofluids*: Digestive fluid extraction; *PAHs in rubber granulate*: Soxlet apparatus; *Metals in rubber granulate*: Microwave-assisted digestion method	Saliva Gastric Fluid Intestinal Fluid	PAHs: HPLC coupled with fluorescence detector Metals: ICP-MS	Except for naphthalene, low PAHs bioaccessibility: below LODs or <3%; Pb bioavailability in the gastric fluid: from 24.7% to 44.3%

Table S2 – Report providing data concerning the bioaccessibility of chemicals from crumb rubber infill material

Reference	Country	Matrix	Markers	Pre-Treatment	Biofluid	Analythical Method	Main Findings
				Grey literature			
**EPA et al. (2019) [17]**	USA	Crumb Rubber	Metals	Biofluid Extraction Acid Digestion	Gastric Sweat + Sebum Saliva	ICP-MS ICP-AES	Metals bioaccessibility decreasing according to the following order: gastric fluids > sweat with sebum > saliva; Zn was the mostly concentrated in the three biofluids, while Mn had the highest bioaccessibility in gastric juices and sweat with sebum.
**Groot et al. (2017) [16]**	Netherland	Crumb Rubber	PAH, BPA Metals, Phthalates Benzothiazoles	Migration Tests Evaporation Test	Sweat Gastrointestinal Juice	n.a.	Ingestion was identified as the most important exposure route; 9% of the PAHs and 20% of phthalates present in rubber granulates were detected in artificial gastrointestinal juices. Metals were also found to be able to migrate.
**Highsmith et al. (2009) [44]**	USA	Crumb Rubber	Pb	Extraction	Digestive fluids	ICP-MS	Pb bioaccessibility ranged from 1.6% to 10.1%
**Lioy and Weisel (2011) [45]**	USA	Crumb Rubber	Metals PAHs SVOC	SVOC: SPME metals: microwave digestion	Sweat Lung Biofluid Digestive Fluid	metals: ICP-MS SVOC, PAHs: GC/MS	PAHs < LODs except acenaphthylene; Metals: Be, Se, As, Ag, and Cd < LOD, Pb, Cu, and Mg mostly concentrated in artificial digestive fluids, V and Cr mostly concentrated in sweat.
**OEHHA (2007) [46]**	USA	Crumb rubber	SVOCs, Metals	Gastric digestion	Gastric fluids	ICP-MS GC-MS	Metals and SVOCs were found to be able to migrate in artificial digestive fluids

Armada D, Martinez-Fernandez A, Celeiro M, Dagnac T, Llompart M (2023) Assessment of the bioaccessibility of PAHs and other hazardous compounds present in recycled tire rubber employed in synthetic football fields. Science of The Total Environment 857:159485 doi:https://doi.org/10.1016/j.scitotenv.2022.159485

EPA, ATSDR, CDC (2019) Synthetic Turf Field Recycled Tire Crumb Rubber Research Under the Federal Research Action Plan FINAL REPORT PART 1 vol 1. U.S. Environmental Protection Agency / Office of Research and Development (EPA/ORD),

Centers for Disease Control and Prevention / Agency for Toxic Substances and Disease Registry (CDC/ATSDR)

Groot GMd, Oomen AG, Mennen MG (2017) Playing sports on synthetic turf pitches with rubber granulate. Scientific background document. National Institute for Public Health and the Environment (RIVM), Bilthoven, The Netherlands

Highsmith R, Thomas KW, Williams RW (2009) A Scoping-Level Field Monitoring Study of Synthetic Turf Fields and Playgrounds. U.S. Environmental Protection Agency, Washington, DC

Kawakami T, Sakai S, Obama T, Kubota R, Inoue K, Ikarashi Y (2022) Characterization of synthetic turf rubber granule infill in Japan: Rubber additives and related compounds. Science of The Total Environment 840:156716 doi:https://doi.org/10.1016/j.scitotenv.2022.156716

Kim H-H, Lim Y-W, Kim S-D, Yeo I-Y, Shin D-C, Yang J-Y (2012a) Health Risk Assessment for Artificial Turf Playgrounds in School Athletic Facilities: Multi-route Exposure Estimation for Use Patterns. Asian Journal of Atmospheric Environment 6(3):206-221 doi:10.5572/ajae.2012.6.3.206

Kim S, Yang J-Y, Kim H-H, Yeo I-Y, Shin D-C, Lim Y-W (2012b) Health Risk Assessment of Lead Ingestion Exposure by Particle Sizes in Crumb Rubber on Artificial Turf Considering Bioavailability. Environ Anal Health Toxicol 27(0):e2012005 doi:10.5620/eht.2012.27.e2012005

Kubota R, Obama T, Kawakami T, Sakai S, Inoue K, Ikarashi Y (2022) Characterization of synthetic turf rubber granule infill in Japan: Total content and migration of metals. Science of The Total Environment 842:156705 doi:https://doi.org/10.1016/j.scitotenv.2022.156705

Lioy PJ, Weisel C (2011) Crumb Infill and Turf Characterization for Trace Elements and Organic Materials. Environmental and Occupational Health Sciences Institute Robert Wood Johnson Medical School, Piscataway, New Jersey

Nishi I, Kawakami T, Sakai S, et al. (2022) Characterization of synthetic turf rubber granule infill in Japan: Polyaromatic hydrocarbons and related compounds. Science of The Total Environment 842:156684 doi:https://doi.org/10.1016/j.scitotenv.2022.156684

OEHHA (2007) Evaluation of Health Effects of Recycled Waste Tires in Playground and Track Products. California Environmental Protection Agency's Office of Environmental Health Hazard Assessment, Sacramento, CA

Pavilonis BT, Weisel CP, Buckley B, Lioy PJ (2014) Bioaccessibility and Risk of Exposure to Metals and SVOCs in Artificial Turf Field Fill Materials and Fibers. Risk Analysis 34(1):44-55 doi:https://doi.org/10.1111/risa.12081

Pronk MEJ, Woutersen M, Herremans JMM (2020) Synthetic turf pitches with rubber granulate infill: are there health risks for people playing sports on such pitches? Journal of Exposure Science & Environmental Epidemiology 30(3):567-584 doi:10.1038/s41370-018-0106-1

Schneider K, de Hoogd M, Haxaire P, Philipps A, Bierwisch A, Kaiser E (2020) ERASSTRI - European Risk Assessment Study on Synthetic Turf Rubber Infill – Part 2: Migration and monitoring studies. Science of The Total Environment 718:137173 doi:https://doi.org/10.1016/j.scitotenv.2020.137173

Zhang J, Han I-K, Zhang L, Crain W (2008) Hazardous chemicals in synthetic turf materials and their bioaccessibility in digestive fluids. Journal of Exposure Science & Environmental Epidemiology 18(6):600-607 doi:10.1038/jes.2008.55
